# A novel less-invasive therapy for a bleeding eroded artery in a giant duodenal ulcer: principles and technical description

**DOI:** 10.1016/j.igie.2022.10.006

**Published:** 2022-11-03

**Authors:** Diogo Turiani Hourneaux de Moura, Bruno Salomão Hirsch, Karina Gondim Moutinho da Conceição Vasconcelos, Luiz Tenório de Brito Siqueira, Saullo Queiroz Silveira, Eduardo Guimarães Hourneaux de Moura, Paulo M. Hoff

**Affiliations:** 1 Instituto D’Or de Pesquisa e Ensino, Hospital Vila Nova Star, São Paulo, Brazil; 2 Gastrointestinal Endoscopy Unit, Department of Gastroenterology, Hospital das Clínicas da Faculdade de Medicina da Universidade de São Paulo, São Paulo, Brazil; 3 Instituto D’Or de Pesquisa e Ensino, Oncologia-Rede D’Or São Luiz, São Paulo, Brazil; 4 Department of Oncology, Instituto do Câncer de São Paulo da Faculdade de Medicina da Universidade de São Paulo, São Paulo, Brazil

## Abstract

**Background and aims:**

Giant duodenal ulcer (GDU) is a challenging condition to manage. Medical and endoscopic therapy often fails, and angiographic embolization can deteriorate GDUs because it may promote ischemia. Surgical treatment is challenging and may increase morbidity. In this article, we describe a promising technique based on the pathophysiology of this condition.

**Methods:**

We report a case of a novel mechanism-based treatment for healing GDU by using modified endoscopic vacuum therapy (EVT). Such therapy promotes macro- and micro-deformation, increases angiogenesis, decreases exudation, and reduces aggressive mucosal factors such as gastric and biliopancreatic secretions.

**Results:**

We describe the case of a 52-year-old man with a history of pancreatic cancer and metastatic disease to the liver who had undergone distal pancreatectomy and left hepatectomy 2 years prior. He was receiving selective internal radiation therapy for right-sided liver metastasis when he was admitted with hypotensive shock after massive GI bleeding. EGD demonstrated a GDU with a large eroded artery, which angiography revealed to be the hepatic artery. Inasmuch as surgery, embolization, and endoscopic vessel-directed therapy were not indicated, modified EVT was performed by use of a triple-lumen tube to allow EVT and nutrition with a single tube. After 3 weeks of therapy, EGD demonstrated healed mucosa, and imaging confirmed no liver ischemia.

**Conclusion:**

Modified EVT is feasible and appears safe and effective for managing complicated GDUs, especially when conventional therapies fail or are not indicated. This strategy may improve the outcomes in patients with GDU, avoiding surgery and reducing morbidity and mortality. Further studies are necessary to confirm our findings.

Giant duodenal ulcers (GDUs) are defined as ulcerations at least 2 cm in diameter, usually involving a large portion of the duodenal bulb. Common presentations are hemorrhage, obstruction, and perforation.^1^ The main cause is peptic ulceration; other causes include Crohn disease, infections, and pancreatic, hepatic, or duodenal cancer.^1^^,^^2^

Recently, with the advances in oncologic treatment, such as selective internal radiation therapy (SIRT) for patients with liver metastasis, adverse events like SIRT-induced peptic ulcers are being reported (approximately 5% of patients) with no consensus for managing this side effect.^3^

Medical and endoscopic treatment of GDU is often unsuccessful. Surgery has been considered the best approach for these patients; however, it is usually challenging because GDUs may adhere to the pancreas, liver, and other structures.^2^ Angiographic embolization may be considered in refractory bleeding before salvage surgery is undertaken. However, it can deteriorate GDUs because it may promote ischemia.^3^, ^4^, ^5^, ^6^, ^7^

In this article, we describe a promising technique based on the pathophysiology of this condition.

## Methods

To our knowledge, this is the first case report of a novel mechanism-based therapy for managing GDU with an eroded artery in a high-risk patient. Such therapy is based on endoscopic vacuum therapy (EVT) , which promotes macro- and micro-deformation, stimulating angiogenesis, decreasing exudation, and reducing aggressive mucosal factors such as gastric and biliopancreatic secretions.^4^, ^5^, ^6^, ^7^ Thus, it may promote healing, especially in ischemic tissue, and might be adapted for treating hemorrhage, improving vascularization, and not increasing ischemia, as is possible with conventional endoscopic vessel-directed therapies and embolization.^5^, ^6^, ^7^

In this case, a modified EVT with use of a triple-lumen tube to allow nutrition and drainage with a single tube through the nares was used. A modified sponge was manufactured on the aspiration lumen of the tube with gauze and incise drape, as previously described by our group.^8^ Then, the distal end of the feeding lumen was positioned in the proximal jejunum and the aspiration portion on the duodenal defect. Finally, the device was connected to a vacuum machine (−125 mm Hg).

## Results

We describe the case of a 52-year-old man with a history of pancreatic cancer and metastatic disease to the liver who had undergone distal pancreatectomy and left hepatectomy 2 years prior. He was receiving SIRT for right-sided liver metastasis when he was admitted with hypotensive shock after massive hematemesis. Laboratory tests showed a hemoglobin level of 4.5 g/dL.

**Figure 1:**
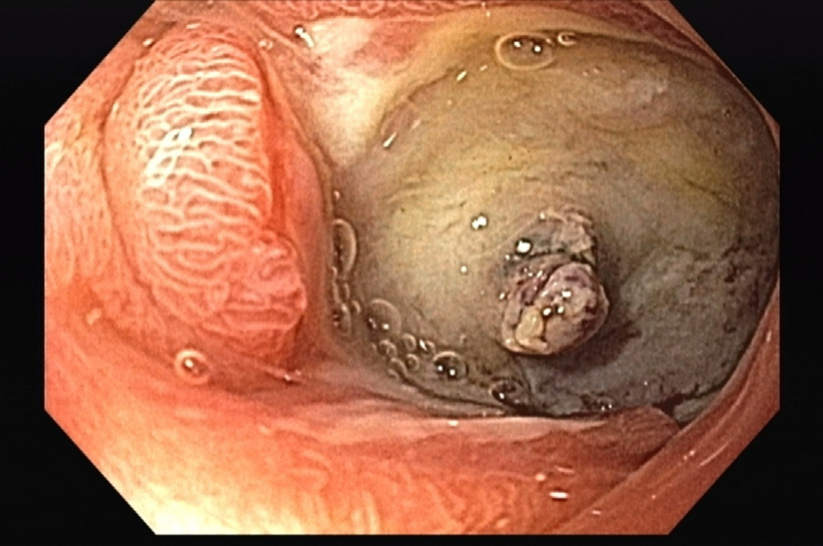
First EGD evaluation demonstrating a giant duodenal ulcer with visible vessel.

**Figure 2:**
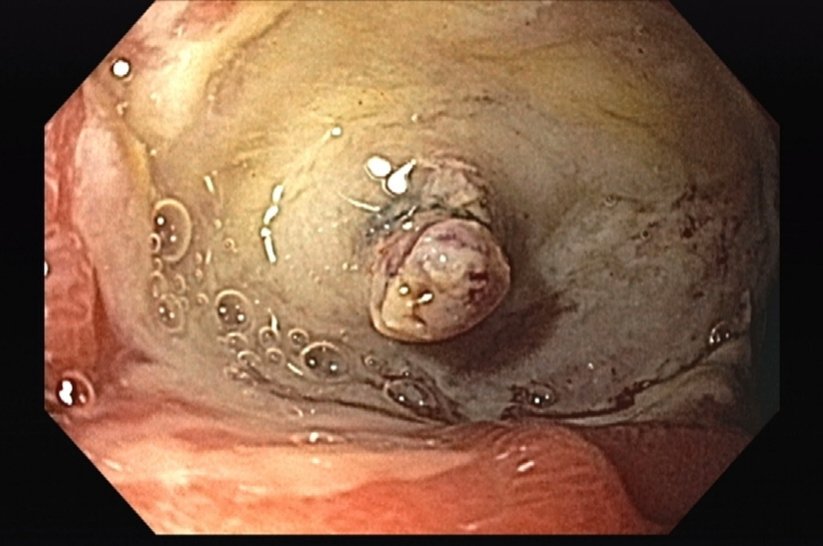
Endoscopic appearance after saline irrigation.

**Figure 3:**
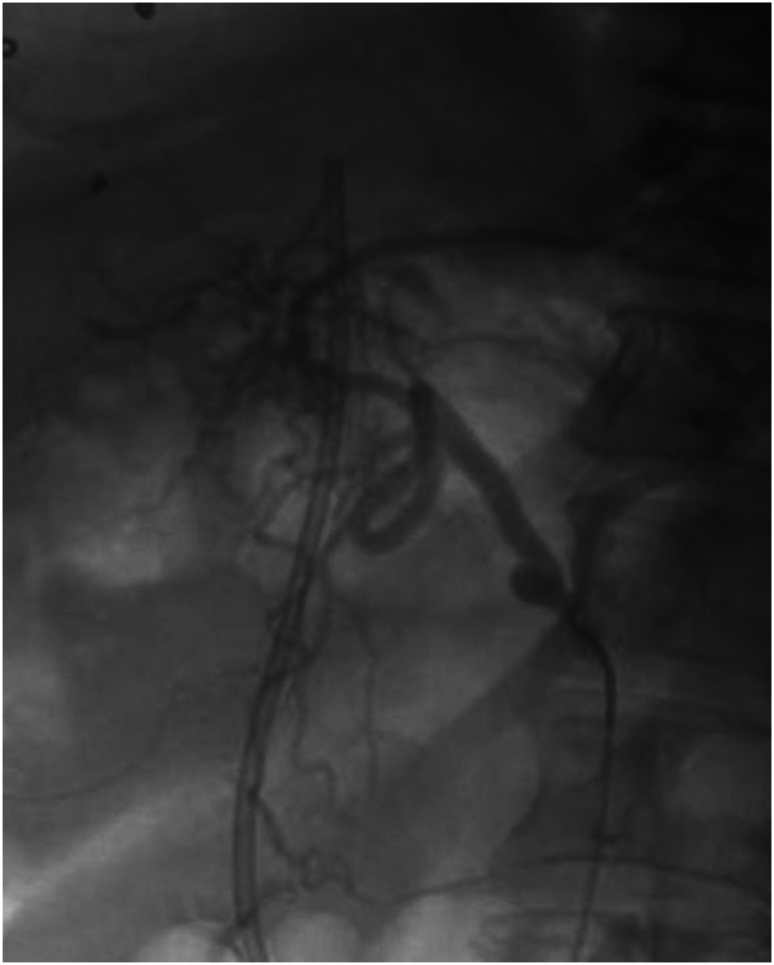
Celiac trunk arteriography revealing irregularities at the distal end of the hepatic artery proper, close to the emergence of the gastroduodenal artery.

EGD demonstrated a GDU with a large eroded artery (Figs. 1 and 2). Owing to the vessel size and the patient’s clinical instability, he was referred for angiographic embolization. However, during arteriography, the visible vessel was identified as the hepatic artery. This was the only blood vessel irrigating the remaining liver because of damage caused by SIRT (Fig. 3). Therefore, embolization and surgery were not indicated, nor was any endoscopic vessel–directed therapy such as over-the-scope-clips and EUS-guided injection of gel foam, cyanoacrylate, or coil. Given that during the procedure there was no active bleeding, hemostatic powder was also not indicated.^9^, ^10^, ^11^, ^12^ On the basis of our experience with the modified EVT for transmural GI defects and diffuse duodenal hemorrhage in patients with severe inflammatory response,^4^, ^5^, ^6^, ^7^ and owing to the low efficacy of high-dose proton pump inhibitors in GDU,^13^ a modified EVT was performed after a multidisciplinary team discussion, including the patient’s family because the procedure was considered an experimental therapy.

**Figure 4:**
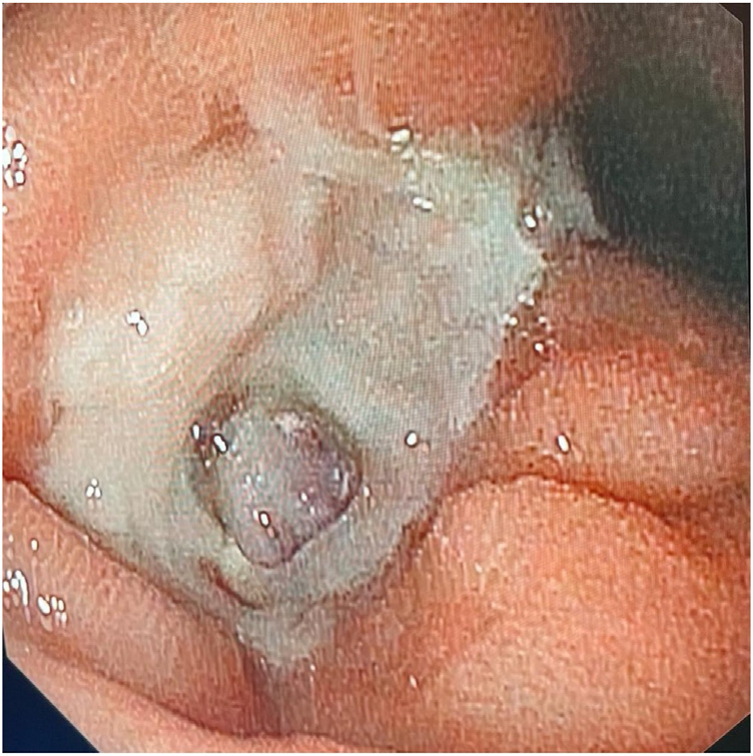
Endoscopic appearance after 1 week of endoscopic vacuum therapy.

**Figure 5:**
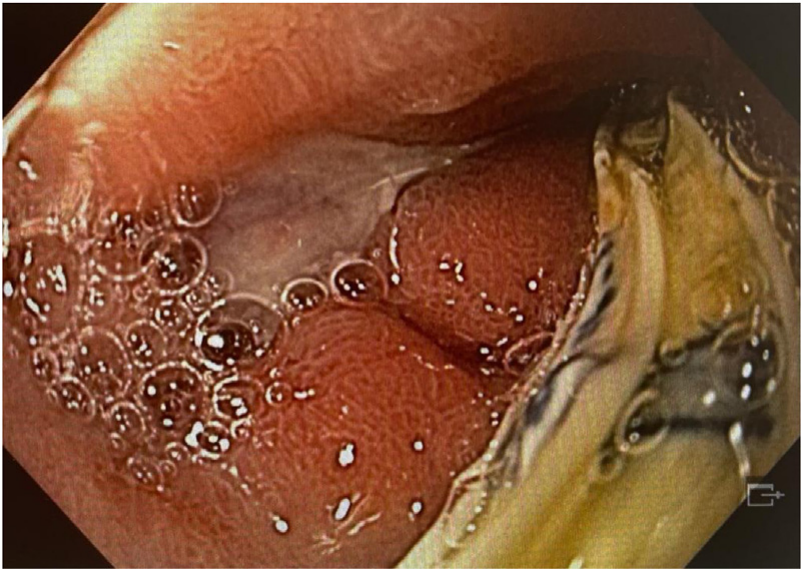
Endoscopic appearance after 2 weeks of endoscopic vacuum therapy.

**Figure 6:**
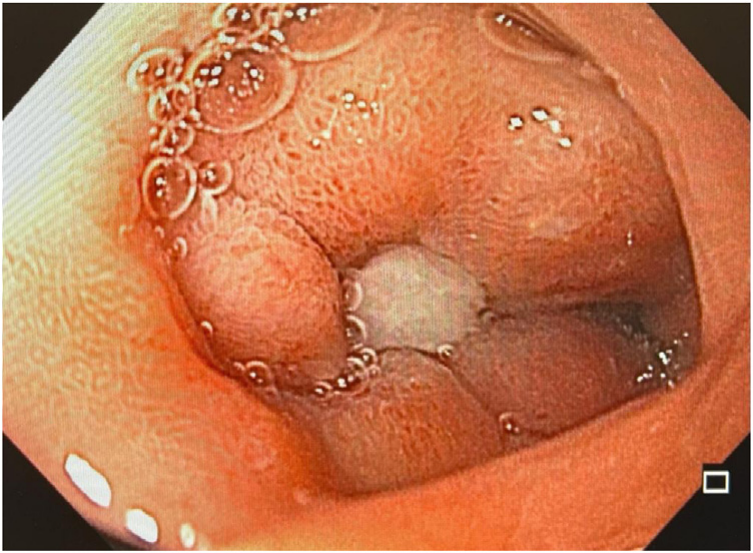
Final endoscopic appearance.

**Figure 7:**
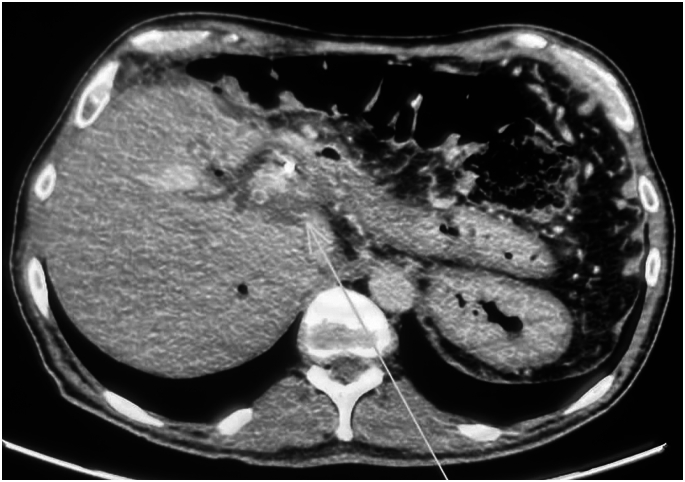
Computed tomography after completion of endoscopic vacuum therapy with no signs of hepatic ischemia.

The patient was successfully treated with the modified EVT system, with the first procedure being followed by 3 weekly EVT system exchanges (Figs. 4 and 5). Follow-up EGD demonstrated healed mucosa with a clean base ulcer (Forrest III) (Fig. 6), and computed tomography showed no signs of liver ischemia (Fig. 7). The patient was discharged 28 days after the first EGD.

## Discussion

Endoscopic treatment remains the criterion standard therapy for managing gastroduodenal ulcer bleeding. However, even novel endoscopic approaches such as over-the-scope-clips, hemostatic powder, endoscopic suturing, and EUS-guided therapies are often ineffective for GDU bleeding.^13^, ^14^, ^15^ Thus, less-invasive approaches are needed, especially for high-risk patients. It is important to emphasize that the EVT does not aim to aspirate blood but to stimulate neoangiogenesis and tissue healing. Additionally, the slippery surface of this modified EVT is not associated with tissue ingrowth, as can occur the traditional polyurethane sponge.^8^

The modified EVT is feasible and appears safe and effective for managing complicated GDUs, especially when conventional therapies fail or are not indicated. This strategy may improve outcomes in patients with GDU, avoiding surgery and reducing morbidity and mortality. Further studies are necessary to confirm our findings.

## Patient Consent

The authors have received appropriate patient consent for the publication of this article.

## Disclosure

*All authors disclosed no financial relationships.*
